# Antibacterial activity and genomic characterisation of a novel *Brevibacillus laterosporus* XJ-24-3 isolated from Xinjiang, China

**DOI:** 10.2478/jvetres-2025-0039

**Published:** 2025-09-17

**Authors:** Ming Wu, Shuang Chen, Guofei Li, Huimei Zhang, Fushuang Duan, Yufei Zuo, Xuepeng Cai, Jie Li, Qingling Meng, Jun Qiao

**Affiliations:** 1 Department of^1^College of Animal Science and Technology, Shihezi University, Shihezi 832003, China; 2 State Key Laboratory of Veterinary Etiological Biology, Lanzhou Veterinary Research Institute, Chinese Academy of Agricultural Sciences, Lanzhou 730046, China

**Keywords:** antibacterial activity, biological characterisation, *Brevibacillus laterosporus*, MRSA, whole genome

## Abstract

**Introduction:**

*Brevibacillus laterosporus* is a significant probiotic with notable antimicrobial activity. This study employs a genome-guided discovery strategy to elucidate the molecular mechanisms and antimicrobial potential of a novel *B. laterosporus* isolate against multidrug-resistant pathogens.

**Material and Methods:**

A *B. laterosporus* strain designated XJ-24-3 was isolated from the soil of a pasture area in Xinjiang, China using the dilution plate method. The XJ-24-3 isolate was identified and characterised by morphology, biochemical properties and 16S rRNA gene sequencing.

**Results:**

The bacterium exhibited bactericidal activity against diverse foodborne pathogens, including methicillin-resistant *Staphylococcus aureus*. Functional genome annotation revealed catalytic activity and molecular binding domains, signifying energy metabolism and environmental adaptability. Carbohydrate-Active Enzyme profiling demonstrated polysaccharide-processing proficiency, corroborated by Cluster of Orthologous Groups classification finding numerous instances of metabolic gene enrichment. Transporter Classification Database analysis indicated the strain predominantly employed active transport mechanisms for substrate translocation. Bioinformatic mining predicted biosynthetic potential for antimicrobial compounds – bogorol A, laterosporulin and linear azol(in)e-containing peptides – positing XJ-24-3 as a promising source of novel broad-spectrum antimicrobial agents.

**Conclusion:**

This study elucidated the genomic characteristics of the novel XJ-24-3 isolate and uncovered the genetic basis for its antibacterial properties, thereby providing a theoretical foundation for the development of novel antibacterial proteins.

## Introduction

Antibiotics play a crucial role in preventing and controlling bacterial infections. However, the widespread and irrational misuse of antimicrobial drugs in the treatment of human and veterinary medicine diseases has led to alarming consequences. This misuse not only undermines the effectiveness of clinical treatments for bacterial infections but also contributes to the rise of multidrug-resistant (MDR) bacteria and exacerbates the already severe threat to global public health (1, 15). In livestock production, the prophylactic and growth-promoting use of antibiotics has strengthened the prevalence of MDR pathogens in farm environments, compromising animal welfare and increasing the risk of zoonotic transmission through the food chain (8). Food-borne pathogens such as *Staphylococcus aureus, Escherichia coli, Salmonella* species and *Listeria monocytogenes* spread worldwide through contaminated food products, and humans and animals are at significant health risk from their spread (2, 36). Of particular concern is the rapid spread of methicillin-resistant *Staphylococcus aureus* (MRSA) and vancomycin-resistant *Enterococcus* (VRE) in recent years (13), which are increasingly detected in livestock and poultry and imply cross-species transmission risks that threaten veterinary therapeutic and public health security (17, 20, 28). Consequently, the frequent acquisition of multidrug resistance by bacteria has rendered many commonly used antibiotics ineffective, presenting a critical challenge to clinical treatment and livestock farming practices (6).

Existing studies have shown that various *Bacillus* species can regulate intestinal flora and improve immune function, and have suggested their suitability as probiotic supplements for both humans and animals (10). In veterinary contexts, *Bacillus*-sourced probiotics have demonstrated potential in enhancing livestock growth performance, reducing enteric pathogen colonisation and mitigating antibiotic dependence (37). In addition, the metabolites of certain *Bacillus* spp. exhibit potent antibacterial (11, 24) and anti-biofilm activity (34), with low effective concentrations and efficacy against drug-resistant strains, making them worthy of investigation as alternatives to traditional antibiotics (31). *Brevibacillus laterosporus* is a Gram-positive bacterium that belongs to the *Brevibacillaceae* family. Studies have demonstrated the antimicrobial properties of *B. laterosporus*. Miljkovic *et al*. (27) successfully isolated and characterised three *B. laterosporus* strains from silage which produced broad-spectrum antimicrobial compounds capable of inhibiting the growth of MDR Gram-positive and Gram-negative pathogens. Cao *et al*. (9) reported the extensive antimicrobial activity spectrum and probiotic potential of *B. laterosporus*. Other research indicated that *B. laterosporus* could produce a diverse range of metabolites with bacteriostatic properties, such as brevilaterins A–E (29), brevibacillin and brevibacillin V (46), bogorol B-JX (19) and bogorols I-L (22), and BL-A60. These metabolites demonstrated significant antimicrobial activity, with notable efficacy against Gram-positive bacteria such as *S. aureus* and *L. monocytogenes* (5).

To develop probiotic products with effective antimicrobial properties for livestock, a novel *B. laterosporus* strain XJ-24-3 was isolated from Xinjiang pastoral soil samples. The strain underwent comprehensive characterisation through integrated morphological, biochemical and molecular analyses, and was investigated for its growth patterns and antimicrobial efficacy. The complete genome of this antimicrobial-proficient isolate was sequenced using hybrid second- and third-generation sequencing platforms.

The strain’s antimicrobial functionality may be exploitable as a sustainable alternative to conventional antibiotics in feed systems. Its application could reduce antimicrobial dependence, enhance food safety and mitigate zoonotic risks. Our findings reveal some of the underexplored potential of environmental microbiomes in combating antimicrobial resistance and represent preparatory work for veterinary medicinal preparation development.

## Material and Methods

### Bacterial strains and culture conditions

The Gram-positive bacteria against which the XJ-24-3 strain of *B. laterosporus* was evaluated in this study were *S. aureus* (ATCC 29213), *L. monocytogenes* (ATCC 19115), *Bacillus cereus* (ATCC 11778) and methicillin-resistant *S. aureus* (MRSA; USA 300). The Gram-negative bacteria tested were *E. coli* (ATCC 25922) and *Klebsiella pneumoniae* (ATCC 10031). Landy’s basal medium, Luria–Bertani (LB) broth, brain heart infusion (BHI) and tryptic soy broth (TSB) were prepared according to established literature protocols. Specifically, *S. aureus* and MRSA were cultured in TSB at 37°C (21), and *L. monocytogenes* (41) and *Bacillus cereus* (35) were cultivated in BHI at 37°C. *Escherichia coli* and *K. pneumoniae* were cultured in LB broth at 37°C with shaking at 180 rpm. *Brevibacillus* species were cultivated in Landy’s basal medium at 32°C with shaking at 200 rpm, as outlined in prior studies (33).

### Isolation and identification of *Brevibacillus laterosporus*

Soil samples collected from pastoral areas in Xinjiang were carefully labelled. A 10 g portion of soil was added to 100 mL of 1% peptone culture solution and incubated at 37°C with shaking at 180 rpm for 1 h. Subsequently, the obtained culture solution was placed in a water bath at 80°C for 30 min. Finally, it was serially diluted and spread onto LB solid culture plates (4), which were incubated at 37°C for 24 h. Gram staining was performed on the wrinkled colonies growing on LB plates, followed by microscopic observation under an oil immersion lens to primarily observe the spore morphological characteristics of the isolated strains. Bacteria with lateral spores were labelled for subsequent processing. To screen for *B. laterosporus* with antibacterial activity, the strains with lateral spores were cultured overnight. The culture was centrifuged at 8,000 rpm for 20 min at 4°C to separate the supernatant, which was filtered through a 0.22-μm membrane to prepare sterile cell-free supernatant. The overnight culture of MRSA in the logarithmic phase was diluted to OD600 ≈ 0.2, and the suspension was spread onto the surface of solid LB medium using a cotton swab. By the agar diffusion method, 100 μL of the cell-free supernatant was added to the wells, while 100 μL of Landy basal medium was used as the negative control. The plates were incubated at 37°C for 24 h, and the diameter of the inhibition zone was measured after incubation. Finally, biochemical identification was performed on the lateral spore-forming bacteria with bacteriostatic activity. The isolates were inoculated into bacterial biochemical reaction tubes, and strain identification was completed based on the results of biochemical tests with reference to *Bergey′s Manual of Systematic Bacteriology* (45). Additionally, the cells of the isolated strains were fixed using 2.5% glutaraldehyde, and morphological observations were conducted using a scanning electron microscope (SEM) (Phenom, Eindhoven, the Netherlands).

### Molecular identification of *Brevibacillus laterosporus*

The specific upstream primer 27F (5′-AGAGTTTGATCCTGGGCTCAG-3′) and downstream primer 1492R (5′-GGTTACCTTGTTACGACTT-3′), designed based on the 16S rRNA gene of bacteria, were employed for the molecular biological identification of the isolated strains. Genomic DNA was extracted from the isolates using a Bacterial Genomic DNA Extraction Kit (TaKaRa Bio, Kusatsu, Japan). The PCR reaction system included 21 μL of water, 1 μL each of the 27F and 1492R primers (0.2 μmol/L), 25 μL of 2× Premix Ex Taq (TaKaRa Bio) and 2 μL of DNA template. The reaction conditions were as follows: pre-denaturation: 95°C for 5 min; followed by 30 cycles, each cycle consisting of denaturation at 95°C for 40 s, annealing at 52°C for 40 s and extension at 72°C for 50 s; finally, a final extension at 72°C for 5 min. The PCR products were analysed using electrophoresis in 1% agarose gel. Subsequently, the amplified fragments were recovered using a DNA recovery kit (TaKaRa Bio) and sequenced. The sequencing results were compared to reference sequences *via* the basic local alignment search tool (BLAST). Finally, MEGA 11.0 software was used to construct a phylogenetic tree to determine whether the isolate belonged to *B. laterosporus* (42).

### Determination of the growth characteristics of the XJ-24-3 isolate

Briefly, the XJ-24-3 isolate was inoculated into fresh Landy’s medium at 1% inoculum and incubated at 37°C for 48 h. At different time points, 200 μL of the bacterial solution was transferred to 96-well plates and placed in an enzyme label reader at 37°C, and the 600 nm wavelength optical density (OD600) value was measured every 3 h for the strain’s growth curve to be plotted. Gram staining was performed on the culture fluids at 8, 16, 24, and 32 h of cultivation, respectively, and the morphological characteristics of XJ-24-3 at different stages were observed under an oil immersion lens. Simultaneously, in the logarithmic growth phase of the XJ-24-3 bacterium in solution, it was inoculated onto 5% sheep blood agar plates and incubated at 37°C for 24 h. The haemolytic activity of XJ-24-3 on the 5% sheep blood agar plates was observed and documented. The isolate was also inoculated into Landy’s basal medium and cultured overnight at 32°C. The OD_600_ value of the bacterial suspension was adjusted to 0.2, corresponding to approximately 10^8^ CFU/mL of live bacteria. A 100 μL aliquot of the suspension was evenly spread onto the surface of LB solid medium, and drug-sensitive paper discs with drug concentrations detailed in Supplementary Table 2 were then placed onto the surface. After overnight incubation at a constant 37°C, the diameter of the inhibition zones was measured, and the strain’s antibiotic sensitivity was analysed.

### Determination of antibacterial activity of the XJ-24-3 isolate

Briefly, the preparation of the sterile supernatant referred to the previous description. For the preparation of the bacterial suspension, the bacterial pellet was washed four times with 1× phosphate-buffered saline (PBS, pH 7.4) with an equal volume of the supernatant. To evaluate the antimicrobial activity of XJ-24-3, an overnight culture of MRSA at logarithmic phase was diluted to an OD_600_ ≈ 0.2 and spread onto solid LB medium using a cotton swab. Three wells were made per Petri dish, into which 100 μL of XJ-24-3 bacterial suspension, cell-free supernatant, and PBS were added respectively. Plates were subsequently incubated at 37°C for 24 h. After incubation, inhibition zone diameters were measured. The antimicrobial spectrum of strain XJ-24-3 was assessed using various Gram-positive and Gram-negative bacteria as indicator strains. To further evaluate the antimicrobial activity, the cell-free supernatant of XJ-24-3 was added to an overnight-cultured MRSA suspension at logarithmic growth phase and incubated at 37°C with 180 rpm shaking. Samples were collected at 0, 1, and 2 h of incubation. The MRSA culture samples were centrifuged, and the harvested MRSA cells were washed and resuspended in PBS (pH 7.4). The cells were subsequently fixed with Glutaraldehyde, 2.5% (EM Grade; Solarbio, Beijing, China) and stored at 4°C overnight. Finally, samples were analysed by SEM to observe the effects of strain XJ-24-3 on MRSA.

### Genome sequencing and gene function annotation

The genomic DNA of the XJ-24-3 isolate was extracted following the protocol described in the Bacterial Genomic DNA Extraction Kit (TaKaRa Bio). Subsequently, whole-genome sequencing of the XJ-24-3 isolate was conducted. The raw sequencing data underwent initial quality control and filtration using FASTP software, ensuring that only high-quality data were retained for downstream analyses (12). Genome assembly and scaffolding were carried out with SPAdes software, utilising the filtered data (32). To enhance the quality of the assembled genome, gaps within the scaffolds were closed using GapFiller software (7). The assembled genome’s quality was then rigorously assessed using QUAST (16) and CheckM (30) software. Additionally, the structural features of the genome were examined by mapping the genome loops of the XJ-24-3 isolate with the IPGA v1.09 online tool (23). Further processing of the whole-genome sequencing results included Gene Ontology (GO) knowledgebase functional annotation and Kyoto Encyclopedia of Genes and Genomes (KEGG) signalling pathway enrichment, Carbohydrate Active Enzyme (CAZy), Cluster of Orthologous Group (COG) and Transporter Classification Database (TCDB) analyses.

### Analysis of secondary metabolite synthesis related gene clusters

The secondary metabolites of the *B. laterosporus* XJ-24-3 isolate were analysed using the antiSMASH online software tool (26). Bacteriocin-related genes in this strain were identified through the BAGEL4 online platform. To further predict bacteriocins within the bacterium’s secondary metabolites, the core peptides and hidden Markov model motifs of the proteins encoded by these genes were examined and analysed (44).

## Results

### Isolation and identification of *B. laterosporus*

**Fig. 1.**
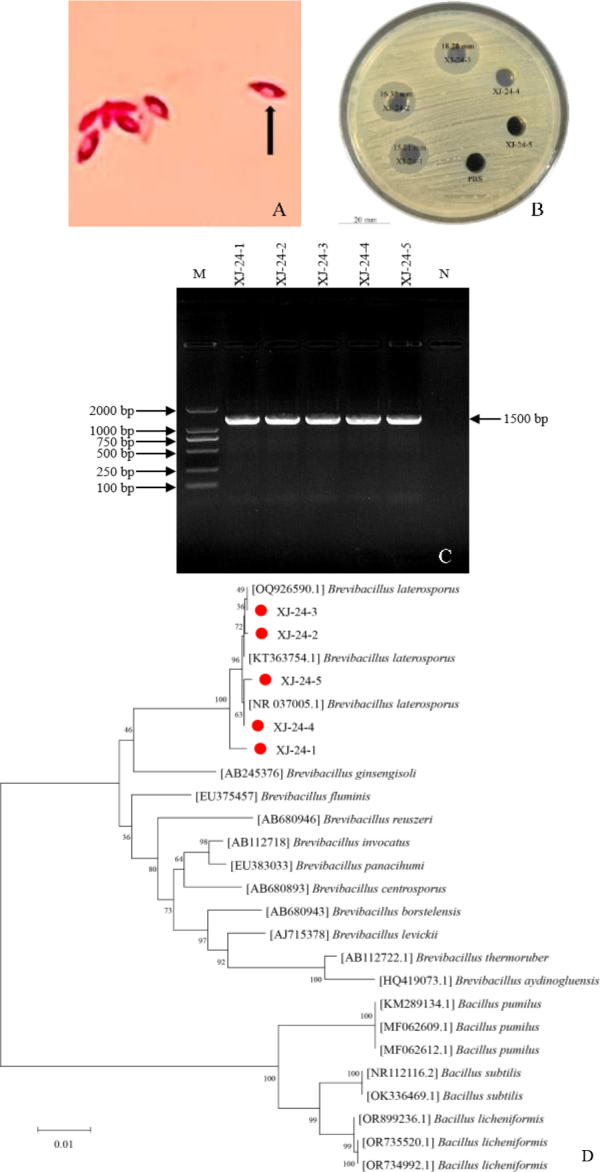
Isolation and identification of *Brevibacillus laterosporus*. (A) Gram staining of five strains of lateral spore-forming bacteria; (B) Antibacterial activity of the sterile supernatants of the culture fluids of five suspected strains; (C) 1% gel electrophoresis of the 16S rRNA of the isolates; M – DNAmarker; N – negative control; (D) Phylogenetic tree constructed based on partial sequences of the 16S rRNA gene. Red circles mark the positions of strains XJ-24-1, XJ-24-2, XJ-24-3, XJ-24-4 and XJ-24-5 isolated in this experiment

Five strains of bacteria with lateral spores (XJ-24-1–5) (Fig. 1A) were successfully isolated from soil samples collected in pastoral areas of Xinjiang. The colony morphology of the five lateral spore-forming bacteria on LB-agar plates was identical, featuring irregular edges, opaque texture, and light yellow colour (Supplementary Fig. 1A). Detection by the agar well diffusion method showed that three strains exhibited antibacterial activity, among which the isolate XJ-24-3 had the strongest antibacterial activity (Fig. 1B). The biochemical identification results of these five lateral spore-forming bacteria were consistent with those of *B. laterosporus* recorded in *Bergey′s Manual of Bacteriology*, preliminarily confirming that the five strains were *B. laterosporus* (Supplementary Table 1). On LB agar medium, the colony morphology of the five bacterial strains was similar to that of *B. laterosporus*, with irregular edges, opaque and light yellow colonies (Supplementary Fig. 1A). Scanning electron microscopy observations showed that bacterial cells presented as fusiform spores with flagella, measuring 2–5 μm in length and 0.5 – 0.8 μm in width (Supplementary Fig. 1B), which was consistent with the description of *B. laterosporus* in *Bergey′s Manual of Bacteriology*. Molecular biological identification results showed that the 16S rRNA of the five lateral spore-forming bacteria all had a size of approximately 1500 bp (Fig. 1C). A phylogenetic tree constructed bythe Neighbor-Joining method based on the 16S rRNA sequences showed that the five strains all clustered with *B. laterosporus* (Fig. 1D). Based on the above findings, the isolated five strains of lateral spore-forming Gram-positive bacteria were identified as *B. laterosporus*. Due to its significant antibacterial activity, XJ-24-3 was selected for subsequent experiments.

### Growth characteristics of *B. laterosporus* isolates

The growth curve results revealed that XJ-24-3 entered the logarithmic growth phase after 3 h of incubation, during which it reproduced rapidly, leading to a significant increase in bacterial density. Following 15 h of incubation, the strain transitioned into the plateau phase (Supplementary Fig. 2). The morphology of XJ-24-3 varied at different culture stages: it appeared as long rod-shaped during the logarithmic phase, short rod-shaped during the stationary phase, gradually formed lateral spores in the decline phase, and eventually developed into complete spores (Supplementary Fig. 1C–F). The haemolytic test results indicated that all three strains of *B. laterosporus* with antagonistic effects in the agar well assay, namely XJ-24-1, XJ-24-2 and XJ-24-3, exhibited no haemolytic activity on 5% sheep blood agar plates (Supplementary Fig. 3). Additionally, XJ-24-3 had growth inhibited by bactericidal drugs, which demonstrated sensitivity to 8 out of the 10 antibiotics tested (Supplementary Table 3).

### *In vitro* antimicrobial activity assay

**Fig. 2.**
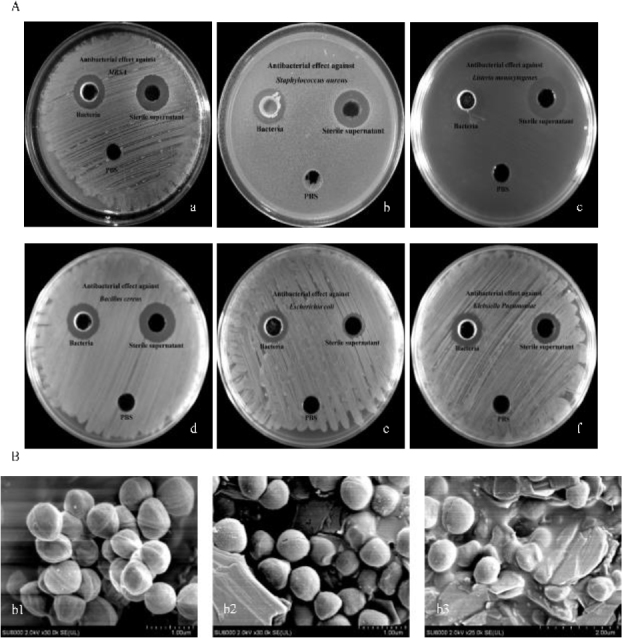
Determination of antibacterial activity of the *Brevibacillus laterosporus* XJ-24-3 isolate. (A) Bacteriostatic activity of bacteria-free supernatant and *B. laterosporus* XJ-24-3 against methicillin-resistant *Staphylococcus aureus* (MRSA), *S. aureus, Listeria monocytogenes, Bacillus cereus, E. coli* and *Klebsiella pneumoniae*; (B) The observation of MRSA treated by cultural supernatant of *B. laterosporus* XJ-24-3 using a scanning electron microscope; b1 – untreated MRSA; b2 and b3 – MRSA treated by the supernatant of *B. laterosporus* XJ-24-3 for 1 h and 2 h, respectively

The supernatant from XJ-24-3 culture supernatant demonstrated strong antibacterial activity against both Gram-positive bacteria such as MRSA, *S. aureus, L. monocytogenes* and *B. cereus*, and Gram-negative bacteria including *E. coli* and *K. pneumoniae* (Supplementary Table 4, Fig. 2A). The inhibitory effect was more pronounced against Gram-positive bacteria. These findings indicate that XJ-24-3 can produce antimicrobial metabolites. Furthermore, the cell-free supernatant derived from the XJ-24-3 strain also exhibited inhibitory activity, forming clear inhibition zones MRSA, *S. aureus, L. monocytogenes, Bacillus cereus, E. coli* and *K. pneumoniae*. Observations through the SEM revealed significant morphological changes in MRSA cells treated with cell-free supernatant. After 1 h of treatment, the cell contours became distorted, and pores began to form on the surface (Fig. 2B b2). Prolonging the treatment to 2 h resulted in intensified cellular damage, with severe collapse of the cell walls and further enlargement of the pores (Fig. 2B b3). In contrast, untreated MRSA cells retained an intact cell wall structure and maintained their characteristic spherical shape (Fig. 2B b1).

### Genome analysis of the XJ-24-3 isolate

**Fig. 3.**
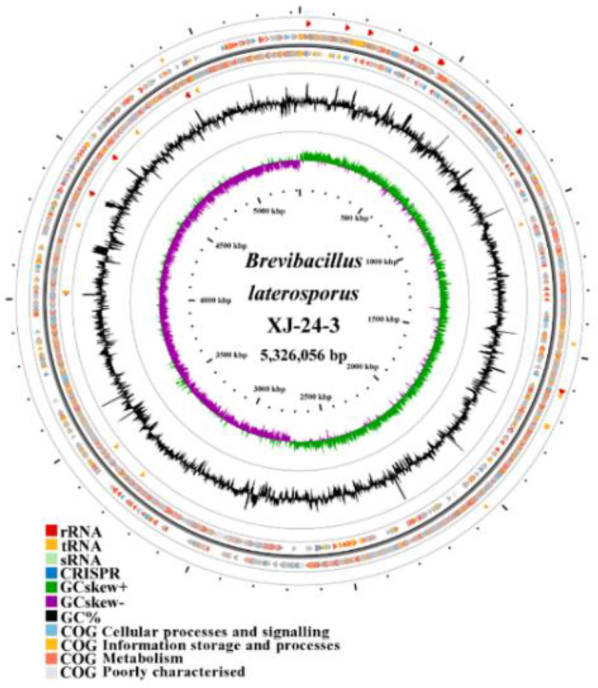
Genome map of the *Brevibacillus laterosporus* XJ-24-3 isolate. Kbp – kilobase pairs; rRNA – ribosomal RNA; tRNA – transfer RNA; sRNA – small RNA; CRISPR – clustered regularly interspaced short palindromic repeats; GC – guanine and cytosine; C G – Cluster of rthologous Groups

The whole genome sequence of the XJ-24-3 isolate was 5,326,056 base pairs (bp) in length, with an N50 length (the sequence length at which 50% of the genome assembly is contained in contigs of this length or longer) of 122,366 bp and a guanine and cytosine content of 40.3%. The number of coding genes was predicted to be 5,039, with a total coding region length of 4,522,398 bp, resulting in an average gene length of 897 bp. This coding region accounted for 84.91% of the total genome sequence. Additionally, 13 genomic islands and 29 prophages were identified. Among the predicted non-coding RNAs, 106 tRNAs and 8 annotated sRNAs were annotated, which are shown in the XJ-24-3 circular genome map (Fig. 3). The genome contained 261 dispersed repetitive sequences.

### Functional annotation analysis of the XJ-24-3 genome

A total of 11,911 genes were annotated through GO enrichment analysis (Fig. 4A). Of these, 6,320 genes (53.1%) were associated with biological processes, primarily involving metabolic activities, cellular functions and localisation. Additionally, 1,196 genes (10.0%) were classified under cellular components, predominantly related to cells and their structural components. In the category of molecular function, 4,395 genes (36.9%) were annotated, with the majority associated with catalytic activity and binding functions. Analysis in the KEGG system revealed 2,012 coding genes involved in various pathways (Fig. 4B). Of these, 1,126 genes enriched metabolic in pathways, with significant enrichment in those related to carbohydrate metabolism (208 genes), amino acid metabolism (205 genes) and cofactor and vitamin metabolism (174 genes). Furthermore, 318 genes were found to operate in environmental sensing pathways, primarily concentrated in membrane transport (174 genes) and signal transduction (143 genes) pathways. An additional 59 genes were involved in pathways such as cellular processes (201 genes), genetic information processing (194 genes) and organismal systems.

**Fig. 4A and B.**
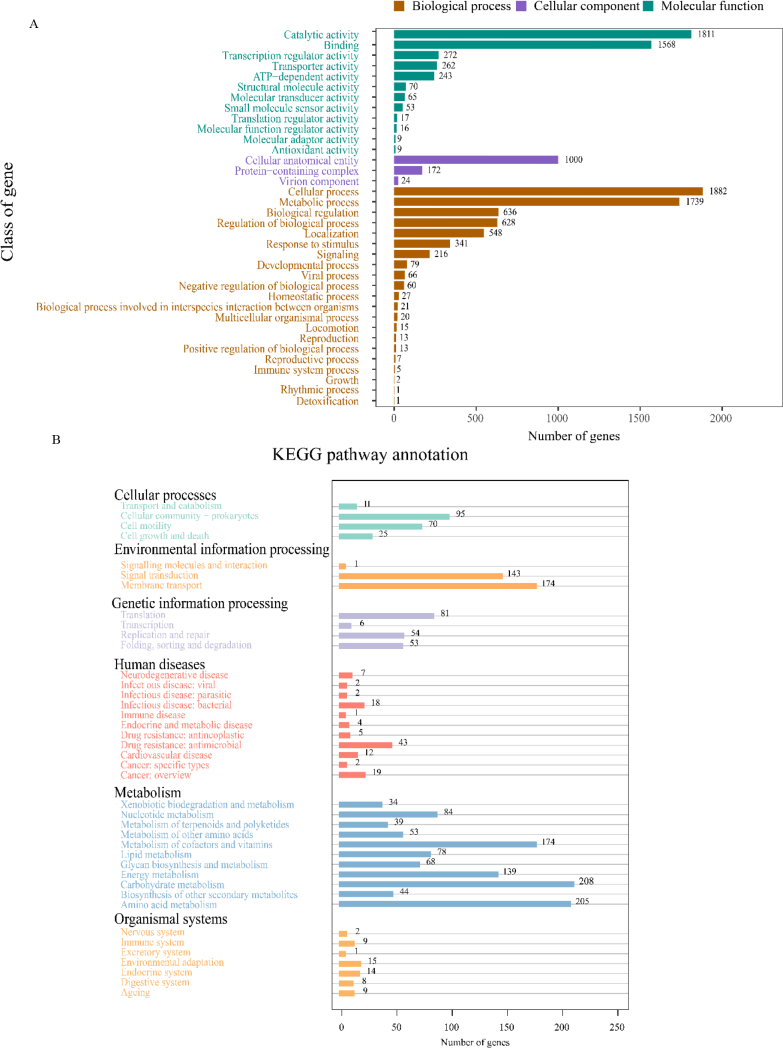
Functional annotation of the genome of the *Brevibacillus laterosporus* XJ-24-3 isolate. (A) GO annotation; (B) KEGG analysis annotation

One-hundred and seventy-one genes associated with carbohydrate-active enzyme families were predicted using the CAZy database (Fig. 4C). Among these, 70 were classified as glycoside hydrolase genes (40.9% of the total), 44 as glycosyl transferase genes (25.7%) and 43 as carbohydrate binding module genes (25.1%). Additionally, 17 carbohydrate esterase genes and 2 oxidoreductase genes were detected, while no polysaccharide lyase genes were found in XJ-24-3. These findings suggest that XJ-24-3 possesses the enzymatic capacity for carbohydrate metabolism through the expression of key enzymes, such as glycoside hydrolases and glycosyltransferases. A total of 3,182 functional genes were annotated through COG analysis (Fig. 4D). Among these, 323 genes (10.2%) were associated with transcriptional regulation, and 362 genes (11.4%) were linked to amino acid transport and metabolism. Additionally, 201 genes (6.3%) were involved in carbohydrate transport and metabolism. The proportions of other functional annotations were as follows: translation, ribosome structure and biosynthesis accounted for 7.29%; the annotation that they were cell wall, membrane and envelope biosynthesis genes was ascribed to 5.75%; signal transduction mechanisms represented 6.69%; coenzyme transport and metabolism made up 6.60%; and inorganic ion transport and metabolism contributed 6.88%. These findings indicate the bacterium’s carbohydrate metabolism, aligning with the results of both the KEGG and CAZy annotations. The TCDB analysis annotated a total of 455 functional genes associated with a primary classification (Fig. 4E). Active transporter protein genes constituted 52.3% of the annotated genes, which is consistent with the membrane transport mechanism’s predominantly relying on active transport processes (39).

**Fig. 4C, D and E.**
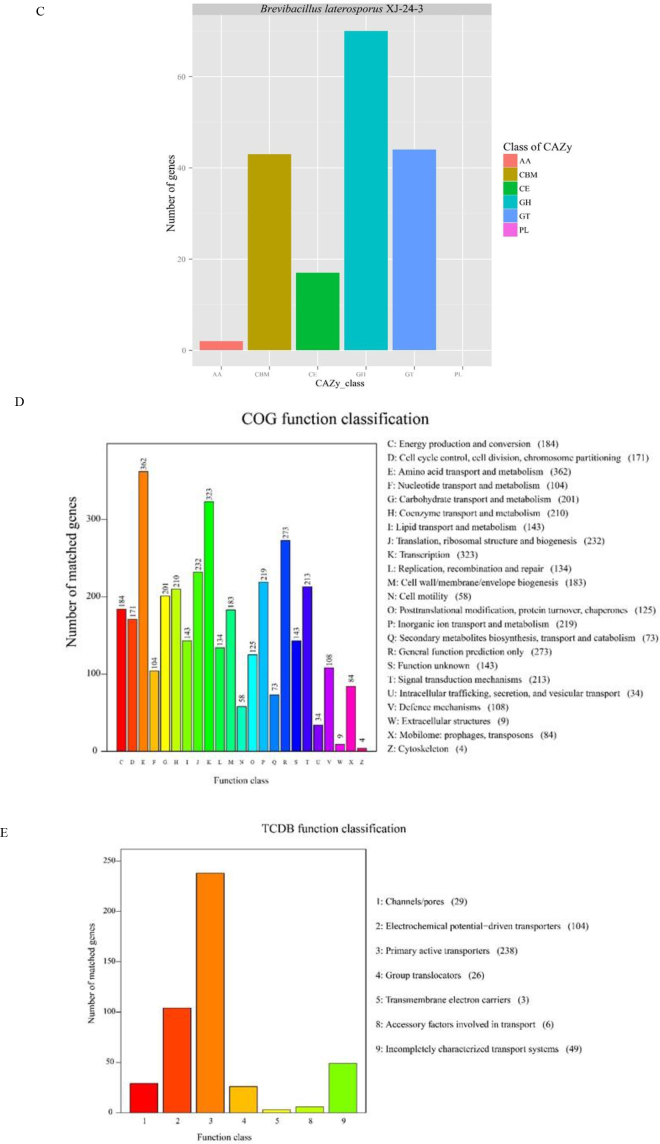
Statistical analysis of secondary metabolic gene clusters in the genome of the *Brevibacillus laterosporus* XJ-24-3 isolate. (C) Carbohydrate-Active Enzyme (CAZy) function prediction; (D) Cluster of Orthologous Groups (COG) analysis; (E) Transporter Classification Database (TCDB) annotation

### Analysis of secondary metabolism gene clusters

**Fig. 5.**
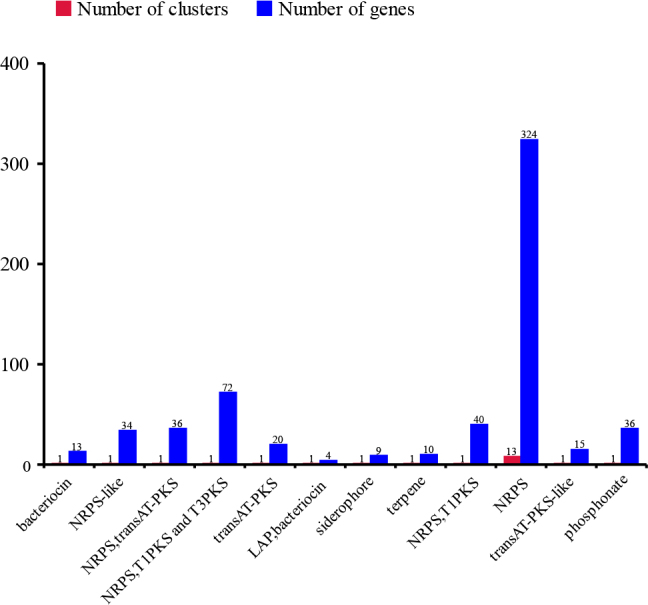
uantities of secondary metabolic gene clusters in the genome of the *Brevibacillus laterosporus* XJ-24-3 isolate

**Fig. 6.**
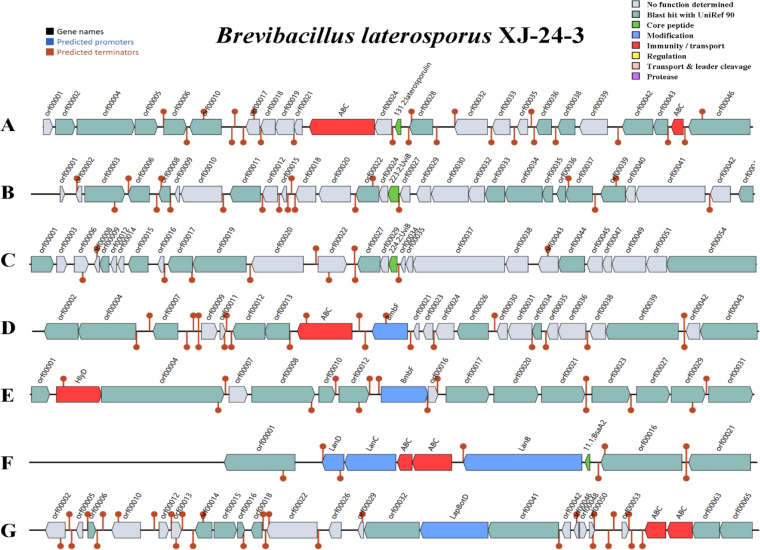
Prediction of bacteriocin-related genes in seven regions (A–G) of the genome of *Brevibacillus laterosporus* XJ-24-3 strain. (A) Lantibiotic ATP-binding cassette (ABC) transporter (ATP – adenosine triphosphate) in red and 131.2 gene (encoding laterosporulin bacteriocin) in green; (B) 223.2 (*uviB*) gene in green; (C) 224.2 (*uviB*) gene in green; (D) Lantibiotic ABC transporter (encoding putative YheS) in red and *bmbF* gene (encoding ribosomal RNA large subunit methyltransferase Cfr) in blue; ( ) Lantibiotic *HlyD* transporter (encoding efflux resistance-nodulation-division transporter permease subunit) in red and *bmbF* gene (encoding radical s-adenosylmethionine domain protein) in blue; (F) Lantibiotic ABC transporter in red and 11.1 (*bsaA2*) gene in green; *lanB, lanC* and *lanD* genes (encoding lantibiotic biosynthesis proteins) in blue; (G) *lapBotD* genes (encoding lantibiotic biosynthesis proteins) in blue and lantibiotic ABC transporter in red

The AntiSMASH-7.0 prediction results show that there are 12 different secondary metabolism gene clusters in the genome of strain XJ-24-3. Among them, the non-ribosomal peptide synthetase (NRPS) gene cluster and the complex hybrid gene cluster with the coexistence of three biosynthesis systems of "NRPS, T1PKS and T3PKS" are the main types. Specifically, the genome mainly contains 17 NRPS gene clusters and 11 gene clusters of other types. It is noteworthy that the results include complex hybrid gene clusters with the coexistence of multiple biosynthesis systems, which have enormous potential in the field of new drug discovery. Among all the predicted genes, the highest number encoded NRPS (324), followed by those encoding the complex hybrid gene clusters with three coexisting biosynthesis systems ('NRPS, T1PKS, and T3PKS') (72) (Fig. 5, Supplementary Fig. 4, and Supplementary Table 5). These 13 distinct biosynthetic pathway types together contained 37 predicted gene clusters associated with secondary metabolite synthesis, including 18 clusters for which no homologous metabolites were identified and 19 clusters associated with known secondary metabolites, such as obafluorin, zwittermicin A, and dipeptide aldehydes, which are synthesised *via* the non-ribosomal pathway (Table 1). The synthetic gene clusters predicted for bogorol A and petrobactin were 100% homologous with their respective known gene clusters in MIBiG database (47). Analysis using BAG L4 predicted seven bacteriocin-producing regions, of which the details are presented in Fig. 6 and shown with the nucleotide lengths of the seven areas of interest in Supplementary Table 6.

**Table 1.** Prediction of molecular sequences with antimicrobial activity in the *Brevibacillus laterosporus* XJ-24-3 isolate

Antimicrobial molecule	Detected by	Amino acid sequence
Laterosporulin	BAGEL4	MACVNECPDAVDDWAYGDWKCHPVEGKYYRHVFAVCMNGANLYCKTEWSKGC
223.2 (UviB)	BAGEL4	MEESVMNALLQQGPFAALFVWLLFSTKKEGRDRETRLVKQAQAREAKLMEHNERMVIQLERNTSTLQQIERSLSGLEMELQELKEKVE
224.2 (UviB)	BAGEL4	MGSFGSLLYLPQGGDKERKSMEEPLFNALLSQGPFAGLFVWLLFSTKKEGRDRETRLVEQAQQREAKLMEHSERMVIQLERNTTTLQQIERSLNGLENELEELKE KVG
Linear azol(in)e-containing peptide	BAGEL4	MDDFQNELKKLRVDKFQGGDVSPWENESQQDAMLVQRRCGRCHHCSCSCSCSCSCSCSCSCSCSCVCLFINCFRCSRCSRCF
Sactipeptide	BAGEL4	MKNYTTPKVKVVNPGVIDVIDSCQCGSKNGAGA
Class I lanthipeptide	BAGEL4	MKKEDLFDLDVQVKEASQAQGDSVVSDLICTTFCSATFCQSNCC
Bottromycin	BAGEL4	MGPVVVFDCMTADFLNDDPNNAELSALELEELESWGVWSEDNQSV
BM1122	antiSMASH	MNKTELIAKVAETSELTKKDATKAVDAVLDAISDALKEGDKVQLIGFGNFEVRERAARKGRNPQTGEEIEIASSKIPAFKPGKQLKDSIK
Lactococcin 972-like	antiSMASH	MDKSQKFPDSPLSKEEWRQLDETIVEMARRQLVGRRFIDIYGPLGEGIQTITN DIYDESRFGNMSLRGESLELTQPSKRVSLTIPIVYKDFMLYWRDMAQARTLG MPIDLSPAANAASSCALMEDDLIFNGNPEFDLPGIMNVKGRLTHIKSDWMES GNAFADIVEARNKLLKMGHSGPYALVVSPELYSLLHRVHKGTNVLEIDHIRN LVTDGVFQSPVIKGGALVATGRHNLDLAIAEDFDSAFLGDEQMNSLMRVYEC AVLRIKRPSAICTLETTEE
Holin	antiSMASH	MKVLFLLHKMRQGGKNGMEESVMNALLQQGPFAALFVWLLFSTKKEGRDR ETRLVKQAQAREAKLMEHNERMVIQLERNTSTLQQIERSLSGLEMELQELKE KVE

## Discussion

The misuse of antibiotics has led to a growing number of bacteria developing resistance to many of these drugs in common use (25). Of particular concern is the rapid proliferation of MDR strains (3), not least because it has caused significant economic losses in the livestock industry. Consequently, identifying effective strategies to prevent and control MDR infections is essential for ensuring the sustainable growth of the livestock sector; these strategies are also equally important for protecting global public health. Among the potential solutions, *Brevibacillus* species have garnered significant attention as a promising focus of research because of their potent antimicrobial activity (19, 38, 47). To contribute to the development of oral probiotic preparations with antibacterial properties for livestock, we isolated a highly bacteriostatic strain of *Brevibacillus laterosporus*, XJ-24-3, and conducted an in-depth analysis of its biological and genomic characteristics.

Various studies have demonstrated that *B. laterosporus* can secrete a variety of secondary metabolites with bacteriostatic effects (22, 27, 43). We analysed the genomic characteristics of a novel isolate of the bacterium and predicted its potential secondary metabolites using AntiSMASH and BAGEL4 software. AntiSMASH analysis revealed that XJ-24-3 harboured a structural gene cluster similar to those known to produce the basiliskamide A, basiliskamide B, laterocidine, ulbactin F, ulbactin G, bogorol A and petrobactin secondary metabolites. This cluster’s similarity to clusters producing bogorol A and clusters producing petrobactin was as high as 100%. Bogorol A, a novel peptide antibiotic derived from *Bacillus maritimus*, exhibited potent antibacterial activity against MRSA and VRE (5). Bogorol A has also been identified in the secondary metabolites of *Brevibacillus laterosporus* (22, 43). BAGEL4 analysis indicated that XJ-24-3 possesses a structural gene cluster producing seven bacteriocins – antimicrobial proteins with bacteriostatic activity. These include laterosporulin, UviB, bottromycin and linear azol(in)e-containing peptides (LAPs). Laterosporulin has been shown to be an immunogenic antimicrobial protein effective against *E. coli* (14, 40). Of particular significance is the presence of a gene in XJ-24-3 encoding the antimicrobial protein bottromycin. This protein is a naturally occurring antibiotic with strong antimicrobial activity against MRSA and VRE.

The *in vitro* bacteriostatic assay demonstrated that XJ-24-3 and its culture supernatant exhibited notable bacteriostatic effects against both Gram-negative and Gram-positive bacteria, with a particularly strong effect on Gram-positive bacteria. The XJ-24-3 isolate displayed significantly stronger bacteriostatic activity compared to species within the *Bacillus* genus which were investigated by *Jia et al*. (18). Furthermore, XJ-24-3 culture supernatant disrupted the cell wall and membrane of MRSA, forming pores on its surface. As exposure time increased, MRSA cells progressively collapsed, and their intracellular contents gradually leaked out. These findings attest to the bactericidal effect of XJ-24-3 on MRSA, suggesting its potential as a promising antibiotic candidate strain.

The genome sequence of the XJ-24-3 isolate was analysed using various approaches, including GO functional annotation, KEGG signalling pathway enrichment, CAZy, COG and TCDB analyses. Gene Ontology analysis revealed that genes associated with catalytic activity and binding functions were the most prevalent. Kyoto Encyclopedia analysis indicated that the strain demonstrated varied energy metabolism capabilities and strong adaptability to environmental changes. Carbohydrate-Active Enzyme and COG analyses highlighted an abundance of carbohydrate enzyme family–related functional genes, suggesting that the XJ-24-3 isolate possessed a significant capacity for carbohydrate metabolism. Transporter Classification Database analysis further revealed that the primary membrane transport mechanism of strain XJ-24-3 likely relied on active transport processes, reflecting the organism’s investment of cellular energy in membrane transport functions.

Summarising, genome-wide analysis revealed that XJ-24-3 was capable of producing a range of broad-spectrum antimicrobial substances, including bogorol A, laterosporulin and LAPs. This genetic characterisation may inform future development of antimicrobial compounds derived from this strain.

## Conclusion

*Brevibacillus laterosporus* XJ-24-3, isolated from Xinjiang soil, exhibited potent antibacterial activity against multidrug-resistant pathogens, including MRSA. Its cell-free supernatant showed broad-spectrum action, particularly against Gram-positive bacteria, forming clear zones of growth inhibition and disrupting MRSA cell wall/membrane integrity. Genomic analysis identified 13 secondary metabolite biosynthetic gene clusters, predicting production of antimicrobial compounds such as bogorol A, laterosporulin and LAPs. Functional annotations revealed robust metabolic pathways, carbohydrate-active enzymes and active transport mechanisms, underscoring its environmental adaptability. Genomic analysis revealed that strain XJ-24-3 contains multiple antimicrobial biosynthetic gene clusters, providing insight into the genetic basis of its antimicrobial activity.

## Supplementary Material

Supplementary Material Details

Supplementary Material Details
