# Supplementary material for: Antibacterial activity and genomic characterisation of a novel Brevibacillus laterosporus XJ-24-3 isolated from Xinjiang, China
Source: J Vet Res. 2025 Sep 17;69(3):313–24. doi: 10.2478/jvetres-2025-0039 (PMC12503217; doi:10.2478/jvetres-2025-0039)
Supplement: Supplementary file 1 — Supplementary Material Details [file jvetres-2025-0039_sm1.pdf]

**Supplementary Table 1.** The biochemical characteristics of *Brevibacillus laterosporus* isolate XJ-24-3

| Physiological or biochemical index | XJ-24-1 | XJ-24-2 | XJ-24-3 | XJ-24-4 | XJ-24-5 |
|---|---|---|---|---|---|
| Anaerobic growth | + | + | + | + | + |
| Voges–Proskauer | – | – | – | – | – |
| Citrate | – | – | – | – | – |
| Propionate | + | + | + | + | + |
| D-xylose | – | – | – | – | – |
| L-arabinose | – | – | – | – | – |
| Gram staining | + | + | + | + | + |
| D-mannitol | + | + | + | + | + |
| Gelatin liquefaction | + | + | + | + | + |
| 7% Growth of sodium chloride | – | – | – | – | – |
| pH5.7 growth | + | + | + | + | + |
| Nitrate reduction | + | + | + | + | + |
| Starch hydrolysis | – | – | – | – | – |
| Lactose fermentation | + | + | + | + | + |

**Supplementary Table 2.** Concentrations of antimicrobials applied to sensitive paper (μg)

| Antimicrobial | Concentration (μg) |
|---|---|
| Tetracycline | 30 |
| Penicillin | 10 |
| Erythromycin | 15 |
| Chloramphenicol | 30 |
| Ampicillin | 10 |
| Ciprofloxacin | 5 |
| Gentamicin | 10 |
| Cotrimoxazole | 25 |
| Ceftriaxone | 30 |
| Lincomycin | 2 |

**Supplementary Table 3.** Determination of drug susceptibility of *Brevibacillus laterosporus* isolate XJ-24-3

| Antimicrobial | Inhibition zone (mm) | Interpretation |
|---|---|---|
| Tetracycline | 21 mm | Susceptible |
| Penicillin | 22 mm | Susceptible |
| Erythromycin | 34 mm | Susceptible |
| Chloramphenicol | 20 mm | Susceptible |
| Ampicillin | 23 mm | Susceptible |
| Ciprofloxacin | 33 mm | Susceptible |
| Gentamicin | 26 mm | Susceptible |
| Cotrimoxazole | 19 mm | Intermediate |
| Ceftriaxone | 25 mm | Susceptible |
| Lincomycin | - | Resistant |

**Supplementary Table 4.** Inhibition diameter of the sterile supernatant of *Brevibacillus laterosporus* isolate XJ-24-3 and of the XJ-24-3 bacterium against different pathogens

| Bacterial species | XJ-24-3 sterile supernatant (mm) | XJ-24-3 bacterium (mm) |
|---|---|---|
| Methicillin-resistant *Staphylococcus aureus* | 17.3 ± 0.2 | 15.8 ± 0.4 |
| *Staphylococcus aureus* | 17.6 ± 0.2 | 16.1 ± 0.3 |
| *Listeria monocytogenes* | 17.2 ± 0.4 | 15.5 ± 0.5 |
| *Bacillus cereus* | 17.2 ± 0.5 | 16.1 ± 0.2 |
| *Escherichia coli* | 11.1 ± 0.5 | 14.1 ± 0.2 |
| *Klebsiella pneumoniae* | 13.1 ± 0.2 | 13.8 ± 0.3 |

**Supplementary Table 5.** Physiological and biochemical characteristics of *Brevibacillus laterosporus* isolate XJ-24-3

| Cluster ID | Type | Most similar gene cluster | Sequence similarity (%) |
|---|---|---|---|
| Cluster 1 | NRPS | obafluorin | 14 |
| Cluster 2 | NRPS | zwittermicin A | 44 |
| Cluster 3 | NRPS-like | N/A | N/A |
| Cluster 4 | Saccharide | N/A | N/A |
| Cluster 5 | NRPS | dipeptide aldehydes | 11 |
| Cluster 6 | Polyketide | basiliskamide A<br>basiliskamide B | 27 |
| Cluster 7 | Polyketide | chejuenolide A<br>chejuenolide B | 7 |
| Cluster 8 | NRPS | laterocidine | 5 |
| Cluster 9 | Saccharide | N/A | N/A |
| Cluster 10 | NRPS | ulbactin F<br>ulbactin G | 85 |
| Cluster 11 | Fatty acid | N/A | N/A |
| Cluster 12 | Saccharide | S-layer glycan | 14 |
| Cluster 13 | NRPS | fengycin | 26 |
| Cluster 14 | Phosphonate | N/A | N/A |
| Cluster 15 | Saccharide | N/A | N/A |
| Cluster 16 | Saccharide | N/A | N/A |
| Cluster 17 | NRPS | bogorol A | 100 |
| Cluster 18 | NRPS | bacillibactin<br>bacillibactin E<br>bacillibactin F | 30 |
| Cluster 19 | NRPS | octapeptin C4 | 11 |
| Cluster 20 | Saccharide | N/A | N/A |
| Cluster 21 | Polyketide | basiliskamide A<br>basiliskamide B | 68 |
| Cluster 22 | Ranthipeptide | N/A | N/A |
| Cluster 23 | NRPS | tyrocidine | 31 |
| Cluster 24 | NRPS | N/A | N/A |
| Cluster 25 | RiPP-like | N/A | N/A |
| Cluster 26 | NRPS | laterocidine | 63 |
| Cluster 27 | NRPS | tauramamide | 40 |
| Cluster 28 | Fatty acid | N/A | N/A |
| Cluster 29 | Other | petrobactin | 100 |
| Cluster 30 | RRE-containing | Pf-5 pyoverdine | 1 |
| Cluster 31 | Fatty acid | N/A | N/A |
| Cluster 32 | NRPS | N/A | N/A |
| Cluster 33 | Fatty acid | N/A | N/A |
| Cluster 34 | RiPP:thiopeptide | micrococcin P1 | 8 |
| Cluster 35 | NRPS | N/A | N/A |
| Cluster 36 | Cyclic-lactone-autoinducer | N/A | N/A |
| Cluster 37 | LAPs | N/A | N/A |

NRPS – non-ribosomal peptide synthase; N/A – there were no homologous metabolites; RiPP-like ribosomally synthesised and post-translationally modified peptide ; RRE – RiPP-precursor-recognition element; RiPP – ribosomally synthesised and post-translationally modified peptide; LAP – linear azol(in)e-containing peptide

**Supplementary Table 6.** Region (area of interest (AOI)) in the genome of *Brevibacillus laterosporus* isolate XJ-24-3

| | AOI | Start nucleotide | End nucleotide | Class |
|---|---|---|---|---|
| A | AOI_01 | 43,383 | 63,536 | 131.2 (laterosporulin) |
| B | AOI_02 | 38,280 | 58,538 | 223.2 (*UviB*) |
| C | AOI_03 | 26,231 | 46,231 | 224.2 (*UviB*) |
| D | AOI_04 | 70,088 | 90,937 | Bottromycin |
| E | AOI_05 | 85,958 | 105,958 | Sactipeptides |
| F | AOI_06 | 120,593 | 140,593 | Class I lanthipeptide |
| G | AOI_07 | 278,099 | 298,099 | Linear azol(in)e-containing peptides |
