# Supplementary material for: Antibacterial activity and genomic characterisation of a novel Brevibacillus laterosporus XJ-24-3 isolated from Xinjiang, China
Source: J Vet Res. 2025 Sep 17;69(3):313–24. doi: 10.2478/jvetres-2025-0039 (PMC12503217; doi:10.2478/jvetres-2025-0039)
Supplement: Supplementary file 2 — Supplementary Material Details [file jvetres-2025-0039_sm2.pdf]

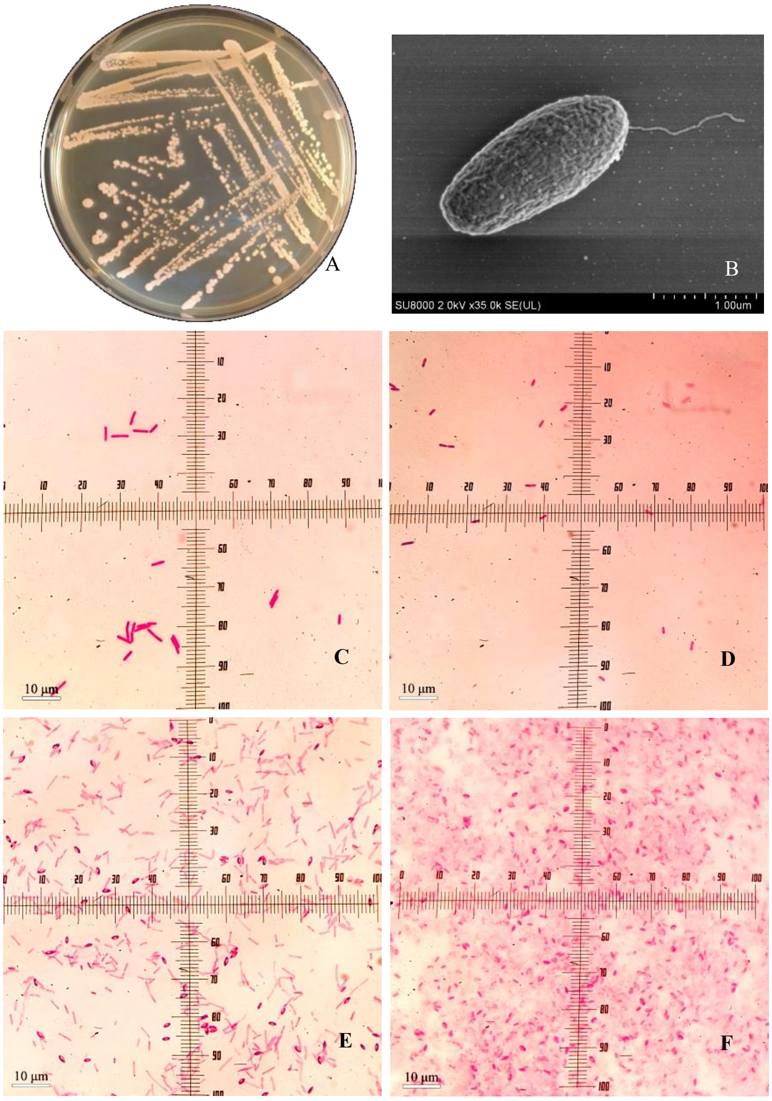

Supplementary Fig. 1. Morphology of *Brevibacillus laterosporus* XJ-24-3 isolate. (A) Colony characteristics of the XJ-24-3 isolate; (B) Spore morphology of XJ-24-3 under scanning electron microscope.; (C–F) Gram staining of the XJ-24-3 strain after 8, 16, 24 and 32 h of cultivation, respectively

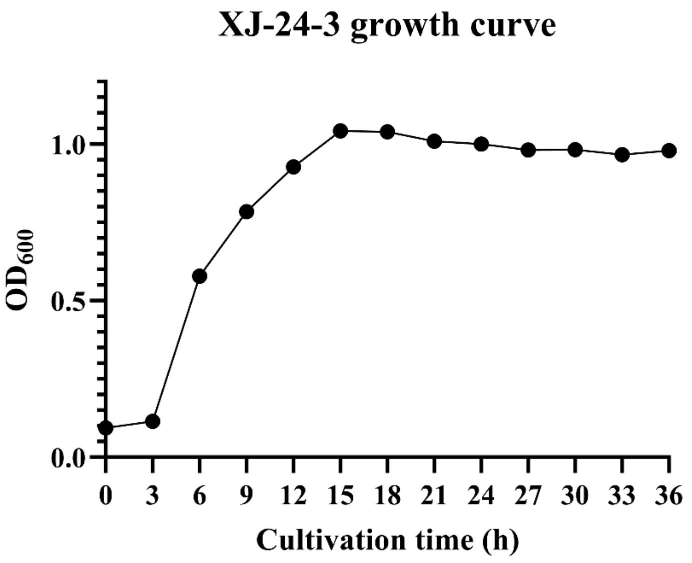

OD_{600} – optical density at 600 nm

Supplementary Fig. 2. Growth curve of *B. laterosporus* XJ-24-3

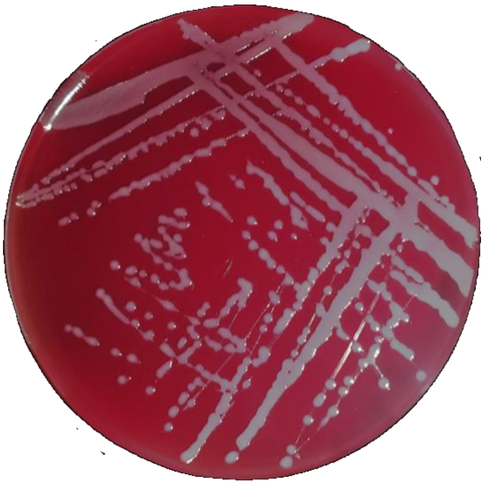

Supplementary Fig. 3. Haemolytic test of *Brevibacillus laterosporus* XJ-24-3 isolate on 5% sheep blood

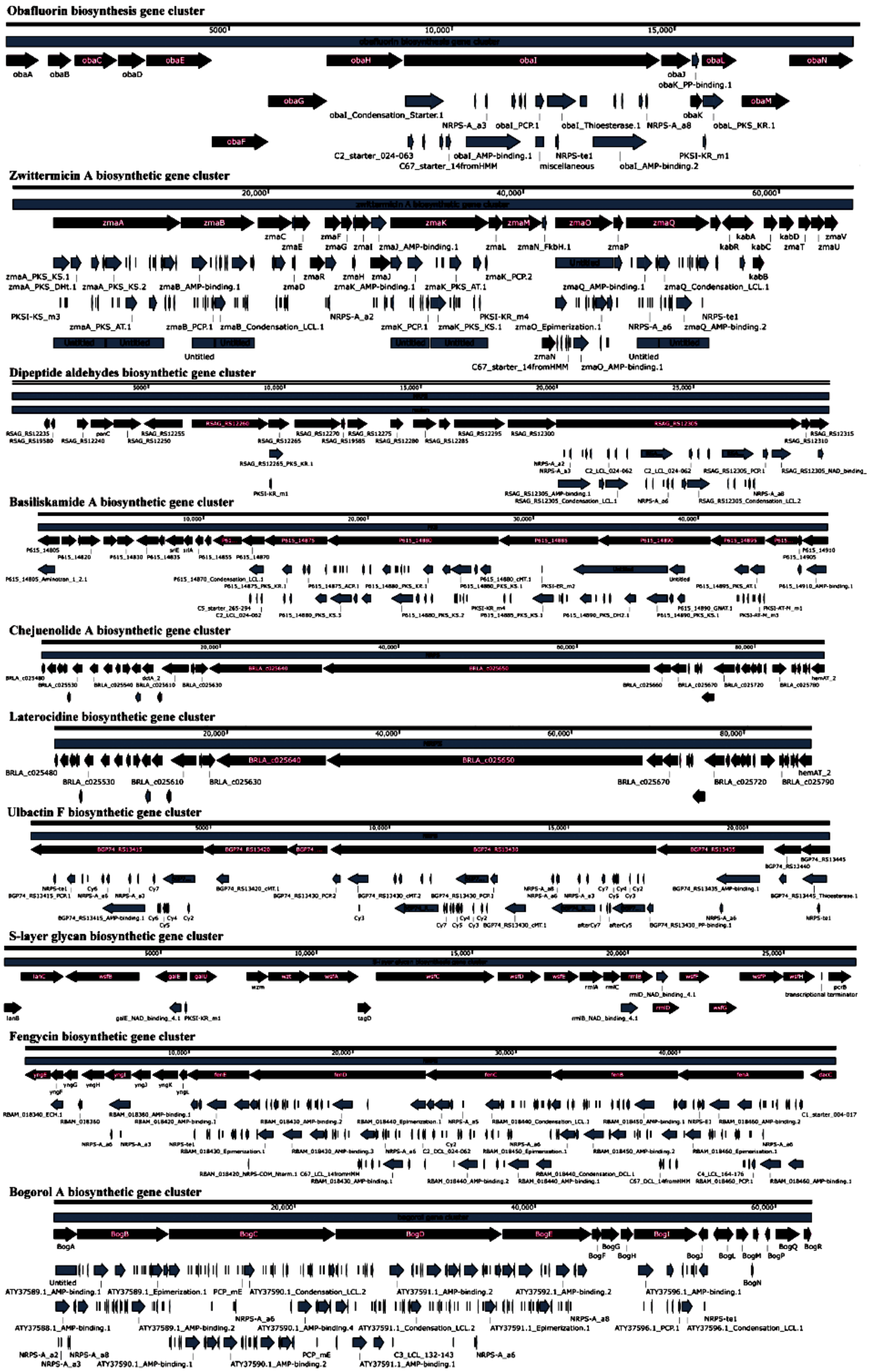
Obafluorin biosynthesis gene cluster
Zwittermicin A biosynthetic gene cluster
Dipeptide aldehydes biosynthetic gene cluster
Basiliskamide A biosynthetic gene cluster
Chejuenolide A biosynthetic gene cluster
Laterocidine biosynthetic gene cluster
Ulbactin F biosynthetic gene cluster
S-layer glycan biosynthetic gene cluster
Fengycin biosynthetic gene cluster
Bogorol A biosynthetic gene cluster

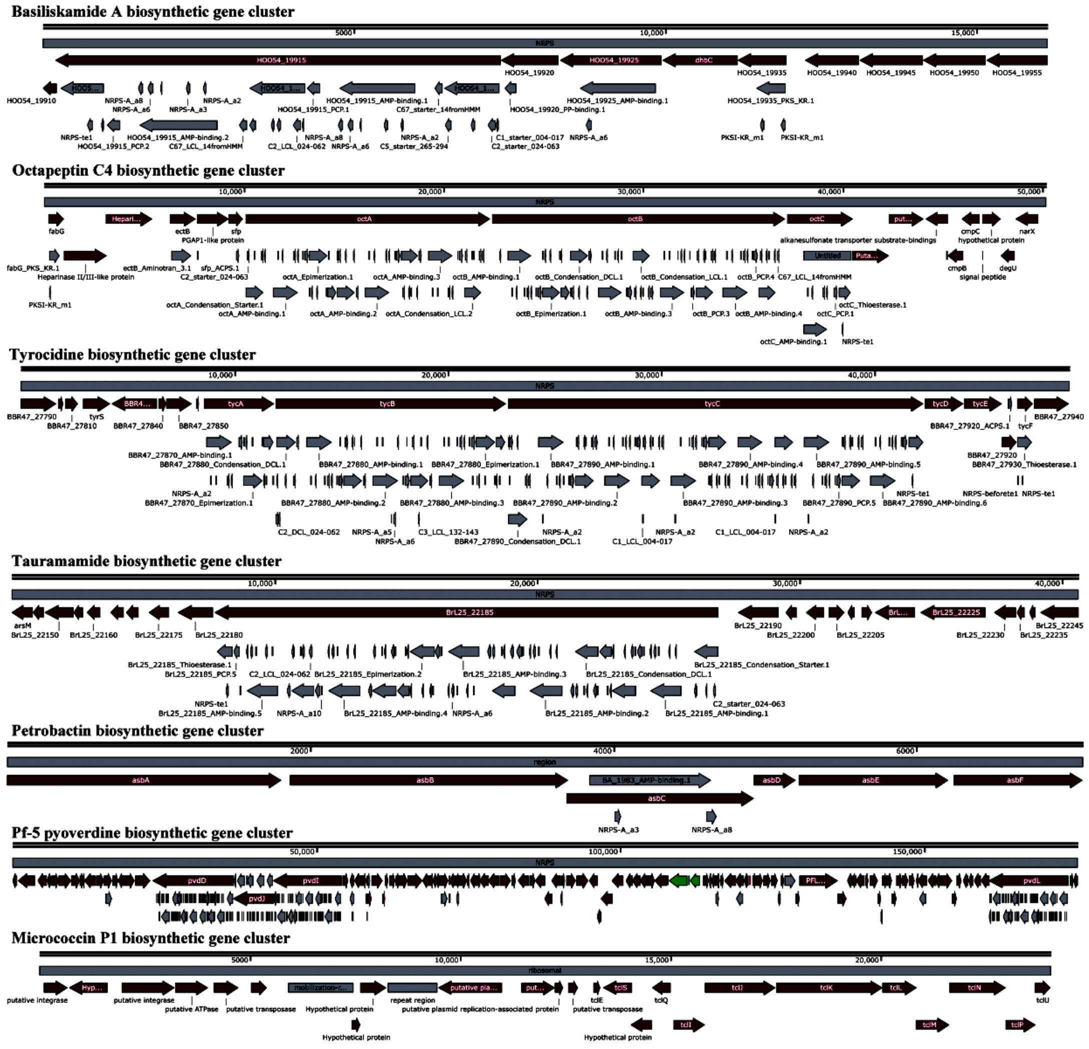

Supplementary Fig. 4. Analysis of gene clusters coding antibacterial substances in the genome of *Brevibacillus laterosporus* XJ-24-3 isolate
